# Sand Painting Generation Based on Convolutional Neural Networks †

**DOI:** 10.3390/jimaging10020044

**Published:** 2024-02-07

**Authors:** Chin-Chen Chang, Ping-Hao Peng

**Affiliations:** Department of Computer Science and Information Engineering, National United University, Miaoli 36003, Taiwan; m0824001@gm.nuu.edu.tw

**Keywords:** neural style transfer, sand painting, morphology, edge detection

## Abstract

Neural style transfer is an algorithm that transfers the style of one image to another image and converts the style of the second image while preserving its content. In this paper, we propose a style transfer approach for sand painting generation based on convolutional neural networks. The proposed approach aims to improve sand painting generation via neural style transfer, which can address the problem of blurred objects. Furthermore, it can reduce background noise caused by neural style transfers. First, we segment the main objects from the content image. Subsequently, we perform close–open filtering operations on the content image to obtain smooth images. Subsequently, we perform Sobel edge detection to process the images and obtain edge maps. Based on these edge maps and the input style image, we perform neural style transfer to generate sand painting images. Finally, we integrate the generated images to obtain the final stylized sand painting image. The results show that the proposed approach yields good visual effects from sand paintings. Moreover, the proposed approach achieves better visual effects for sand painting than the previous method.

## 1. Introduction

Sand painting [1] is an art in which colored sands and other powdered pigments are splashed onto the surface of a work to form fixed or unfixed sand paintings. In a fixed sand painting, an artist scatters sand into a container to create an artwork. For unfixed sand paintings, the artist performs the above during the ceremonial stage. Sand painting is a classical form of artistic expression. However, the existing approaches for generating artwork primarily focus on oil, watercolor, and other types of paintings. In recent years, sand painting has emerged as a unique and innovative type of art that is loved by performers and audiences. Sand painting performance has evolved from merely animation to being combined with painting, lighting, music, and other effects, to provide the audience with enjoyable visual stimulation. However, maintaining sand painting equipment is difficult, and creating sand paintings is costly; thus, the learning of sand painting and the spread of sand painting artworks are hindered.

Owing to the rapid development of modern science and technology, artificial intelligence has been utilized increasingly, and applications related to artificial intelligence have increased. Machine learning is a branch of artificial intelligence, and deep learning is a branch of machine learning. Deep learning is the fastest growing branch of artificial intelligence. It simulates the operation of human neural networks and uses numerous artificial neurons to perform calculations. Owing to the acceleration of hardware and parallel computing technologies, the application of deep learning in image processing has become increasingly common, and style transfer has garnered significant attention. Image style transfer is a technology used to transfer image styles. Its main concept is to specify an input image as a content image and another image as a style image. The style transfer approach transfers the image style while ensuring the style of the content image and obtains the final output of a stylized image. The style of an image can be the work of a certain artist or the style of images captured by an individual. Style transfer approaches have been investigated extensively. Previous methods could only process the synthesis of colors and textures in a simple manner. Meanwhile, abstract artistic styles must be targeted and processed differently in one style. Owing to recent developments in deep learning, researchers have developed methods that use neural networks to manage style transfers. These methods are universal and can manage difficult style transfers easily.

Several approaches for the style transfer [2] and simulation of sand painting have been investigated intensively [3,4,5,6,7,8,9,10,11,12]. Li et al. [3] proposed a neural style transfer approach based on the VGG-16 model [4]. They used advanced features to generate sand paintings based on the content and style features of input images. Their approach can achieve efficient sand painting style transfers; however, the transfer effect is unclear. Wu et al. [5] proposed an algorithm based on cloud models for uncertain strokes in interactive sand paintings. Their approach improved the ordinary cloud model and enabled the development of an interactive painting algorithm with uncertain strokes. Moreover, they used different parameters to draw various styles of sand paintings. However, their approach can only be used to draw text strokes and not for style transfer of an entire image. Chen and Wong [6] proposed a real-time auto-stylized sand art drawing system. The contours of the image were computed and converted into strokes. Subsequently, these strokes were detected and categorized based on geometric rules. The sand painting style conversion effect of this approach is favorable and allows one to view the drawing process in real time. However, it is only suitable for simple images without backgrounds. Song and Yoon [7] proposed an approach that replicates the sand painting drawing process. First, the outline and stroke features of an input image were computed; next, a drawing algorithm was used to draw in the image space. Although the achieved effect was favorable, the freedom offered was limited as the image can only be converted to a preset single style. Moreover, the details could not be fully preserved. Yang et al. [8,9] proposed a sand painting simulation algorithm based on Kinect, which is a colored sand painting simulation based on detail preservation, and a multitouch sand painting simulation system [10]. However, their approach did not provide a function for transferring existing images into sand painting styles. Yang et al. [11] proposed a self-adaptive approach to generate sand paintings in real time. They used the height field to simulate sand flow and rapidly obtained a generated sand painting. However, they used a simple light model in their approach, which resulted in insufficient shadows. Moreover, the effect of the generated colored sand painting was not satisfactory. Zou et al. [13] presented an algorithm to generate styled neural painting images using image-to-painting translation techniques. They proposed a neural rendering algorithm to emulate the vector renderer behavior and predict strokes. Their approach successfully generated the desired paintings using global appearance and local texture methods with a high degree of fidelity. Zhu et al. [14] presented a sand painting conversion approach for creating art styles and preserving details. This approach can be classified into two modules: coarse waving for an entire image and detail preservation for specific areas. In their approach, realistic sand paintings were generated automatically without user intervention. Experimental results revealed the effectiveness of their approach in converting sand painting-style images.

In this paper, we propose an approach to fulfill the main objectives of improving sand painting generation using neural style transfer based on convolutional neural networks (CNNs). First, we segment the objects from a content image. Next, we perform morphological operations on the content image to obtain smooth images. Third, we use an edge detector to process the images and obtain edge maps. For these edge maps and the input style image, we perform neural style transfer to generate sand painting images. Finally, we integrate the generated images to obtain the final stylized sand painting image. Because only low-level image features at the pixel level are used, the generality of conventional methods is unsatisfactory and considerable manual tuning is typically required. Our approach uses the advanced features of an image and automatically generates a sand painting by separating and reorganizing the content and style features. The contributions of our approach are as follows:

- We propose an approach for sand painting generation via style transfer.
- The proposed approach can retain the details of the main objects and improve the stylized rendering of the sand painting.
- The proposed approach can reduce the background noise caused by neural style transfer.

This paper is structured as follows: Section 2 provides an introduction to neural style transfer and the associated architectural formulas. Section 3 describes the proposed approach, including main object segmentation, morphological image processing, edge detection, and sand painting generation. Section 4 presents the experimental results and discussion, including the implementation details for the experiments, the used content and style images, and comparisons. Finally, Section 5 presents the conclusions and directions for future research. 

## 2. Neural Style Transfer

The main purpose of texture transfer is to synthesize textures from a source image to preserve the semantic content of the target image. Most texture transfer approaches use various techniques to preserve the structure of the target image. However, they use only low-level image features of the target image to perform texture transfer. Neural style transfer is a technique that transfers the style of one image to another image, thus providing the style of the other image and preserving its content. Hence, neural style transfer from a reference image to a target image can be solved using a texture transfer technique that constrains a texture synthesis algorithm using feature representations from CNNs. Gatys et al. [15] proposed a neural style transfer technique to transfer the style of one image to another image using CNNs to train generic feature representations. These representations can be used to independently process and manipulate the content and style of an image. The neural style transfer technique was based on the VGG-19 model [3]. However, the VGG-19 model does not use any fully connected layer and uses average pooling instead of maximum pooling.

To understand the neural style transfer better, we define a content image $x_{c}$ and a style image $x_{s}$. In layer l of a CNN, $F_{l}$ is the vectorized feature map of the layer, $N_{l}$ is the number of feature maps in the layer, and $M_{l}$ is the product of the height and width of the feature map in the layer. Neural style transfer generates a new image $\hat{x}$ by minimizing the loss function of the content image $x_{c}$ and style image $x_{s}$.

The loss function of the new image $\hat{x}$ is defined by jointly minimizing the distance between the feature representations of the content image and the style image of the CNN.

(1) $$L_{total}=\alpha L_{content}+\beta L_{style}$$

where $L_{total}$ is the total loss function, $L_{content}$ is the content image loss function, $L_{style}$ is the style image loss function, and α and β are the weights of the content image and style image, respectively.

In the network, the higher layers capture the high-level content, and the lower layers capture the exact pixel values of the input image. Each network layer defines a nonlinear filter bank. The content image $x_{c}$ is encoded in each layer of the CNN using filter responses. The content image loss function in layer $l_{c}$ is defined as the squared error loss between two feature representations, as follows:

(2) $$L_{content}=\frac{1}{N_{l_{c}}M_{l_{c}}(x_{c})}\sum_{ij}{\left(F_{l_{c}}\left(\hat{x}\right)-F_{l_{c}}\left(x_{c}\right)\right)}_{ij}^{2}$$

where ${F_{l_{c}}\left(x\right)}_{ij}$ denotes the activation of the *i*th filter at position *j* in layer $l_{c}$ in response to x.

A feature space can be used to capture texture information to obtain a representation of the style of a sand painting. The feature space can be constructed using the correlations between the different filter responses in each layer of the CNN. A feature space that can capture the style information is constructed on top of the filter responses in any network layer. For a style image $x_{s}$, the style image loss function is defined by minimizing the mean-squared distance between the entries of the Gram matrices from the input style image and those of the generated sand painting, as follows:

(3) $$L_{style}=\sum_{l}\frac{1}{4N_{l}^{2}}w_{l}\sum_{ij}{\left(G_{l}\left(\hat{x}\right)-G_{l}\left(x_{s}\right)\right)}_{ij}^{2}$$

where ${G_{l}\left(x\right)}_{ij}$ is the Gram matrix of feature maps *i* and *j* in layer l in response to image x. Based on [13], we used the VGG-19 [1] model and included “conv4_2” as the layer $l_{c}$ for the image content, as well as Gram matrices from layers “conv1_1”, “conv2_1”, “conv3_1”, “conv4_1”, and “conv5_1” as the image statistics of the model style.

## 3. Proposed Approach

Our approach is intended to improve sand painting generation via neural style transfer, which can manage the issue of object blurring. Furthermore, our approach can reduce background noise caused by neural style transfers. A sand painting comprises several strokes. We transform the content image into a grayscale image and detect the edges of the grayscale image as stroke features. Moreover, to obtain a smoother style transfer effect, we utilize morphological image processing techniques. 

**Figure 1:**
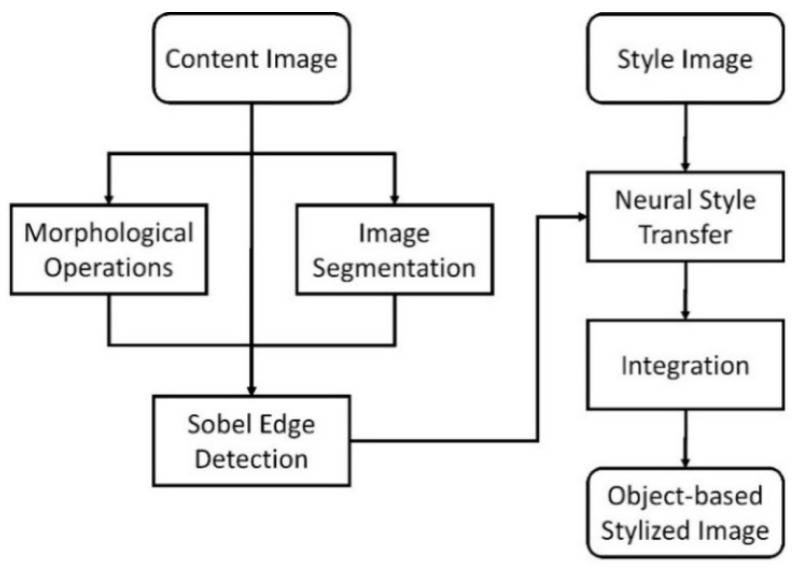
Flowchart of our approach.

A flowchart of the proposed approach is shown in Figure 1. First, we input the content image. Subsequently, the main objects are segmented from the content image. Morphological operations are performed on the content image to obtain smooth images. Next, we perform Sobel edge detection to process the images to obtain the edge maps. For these edge maps and the input style image, we perform neural style transfer to generate sand painting images. Finally, we integrate the generated images to obtain the final object-based stylized sand painting image.

### 3.1. Main Object Segmentation

In our approach, we segment the main objects and integrate them with different weights to emphasize the effect of the main objects [16]. We use the GrabCut algorithm [17] to segment the main objects from the content image because it can effectively distinguish the foreground and background of the image, as well as segment them. 

**Figure 2:**
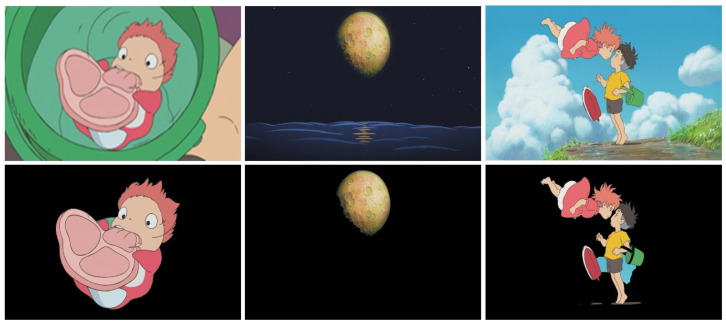
Results of main object segmentation. Images shown on the top row are input content images, and the bottom row are images with segmented main objects.

The GrabCut algorithm only requires the user to specify the foreground area in which the object to be segmented is located. It can automatically calculate the foreground and segment the result. This algorithm first requires the user to mark a rectangular foreground area containing the object to be segmented in the image. Subsequently, it uses the remaining background data to distinguish between the foreground and background areas within the foreground area. Finally, it uses a Gaussian mixture model to model the foreground and background and segments the objects in the foreground. Figure 2 shows the results of the main object segmentation. Images shown on the top row are input content images, and the bottom row are images with segmented main objects. 

### 3.2. Morphological Image Processing

We perform morphological image processing to achieve a smoother style transfer [18]. The basic operators of morphological operations are dilation, erosion, closing, and opening. In the proposed approach, the closing and opening operations are used to process the content image to remove noise. Closing refers to dilation followed by erosion, whereas opening refers to erosion followed by dilation. The result of image A dilated by a structuring element B can be written as $D(A,B)=\underset{b\in B}{⋃}A+b$. The erosion of image A by a structuring element B can be written as $E(A,B)=\underset{b\in -B}{⋂}A+b$. 

**Figure 3:**
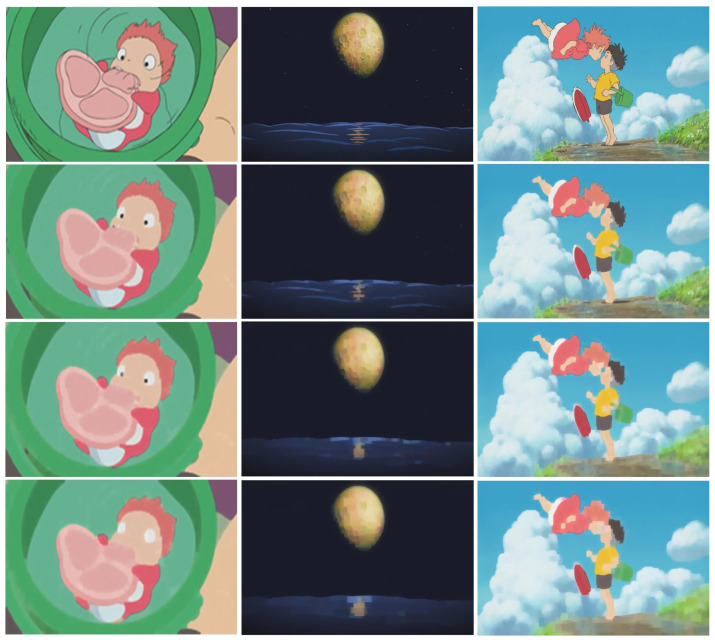
Morphological image processing results. Images shown on the top row are input content images and images shown on the second row to the bottom row are the three smoothed results using 5 × 5, 9 × 9, and 13 × 13 structuring elements, respectively.

In the proposed approach, a content image was used as the input image. Subsequently, three square structural elements of various sizes were employed to perform closing and opening computations on the input image and obtain three layers of smooth images. Structure elements measuring 5 × 5, 9 × 9, and 13 × 13 were used to obtain multiple results. Figure 3 shows the smoothed results of the morphological image processing. Images shown on the top row are input content images and images shown on the second row to the bottom row are the three smoothed results using 5 × 5, 9 × 9, and 13 × 13 structuring elements, respectively.

### 3.3. Edge Detection

The structure of a sand painting comprises several different strokes. First, we transformed the content image into a grayscale image. Subsequently, we detected the edges of the grayscale image as the stroke features. We compared four edge detection methods [19], namely, the Sobel operator [20], Canny edge detector [21], extended difference of Gaussian (XDoG) [22], and holistically nested edge detection (HED) [23]. A comparison of the sand painting effects of these edge detection methods showed that the Canny edge detector did not preserve the features of the objects. Meanwhile, the XDoG and HED showed extremely strong backgrounds. The Sobel detector detected the edges of the main objects and the background more evenly. Therefore, we used the Sobel detector to detect the edges. 

The Sober operator is a small integer filter that uses the horizontal and vertical gradients in the image as the basis for judgment. The two filters are convolved with the image. When the gradient value is greater than the threshold, it is regarded as an edge. Let the input image be *A*. The horizontal and vertical operators are used to obtain the horizontal and vertical gradients of the image, respectively. The Sobel operators in the *X* and *Y* directions are expressed as

(4) $$G_{x}=\left[\begin{array}{ccc} +1 & 0 & -1\\ +2 & 0 & -2\\ +1 & 0 & -1 \end{array}\right]\times A,\;\text{and}$$

(5) $$G_{y}=\left[\begin{array}{ccc} +1 & +2 & +1\\ 0 & 0 & 0\\ -1 & -2 & -1 \end{array}\right]\times A.$$

The gradient magnitude at each pixel is computed as follows:

(6) $$G_{gradient}=\sqrt{({G_{x})}^{2}+({G_{y})}^{2}}.$$

**Figure 4:**
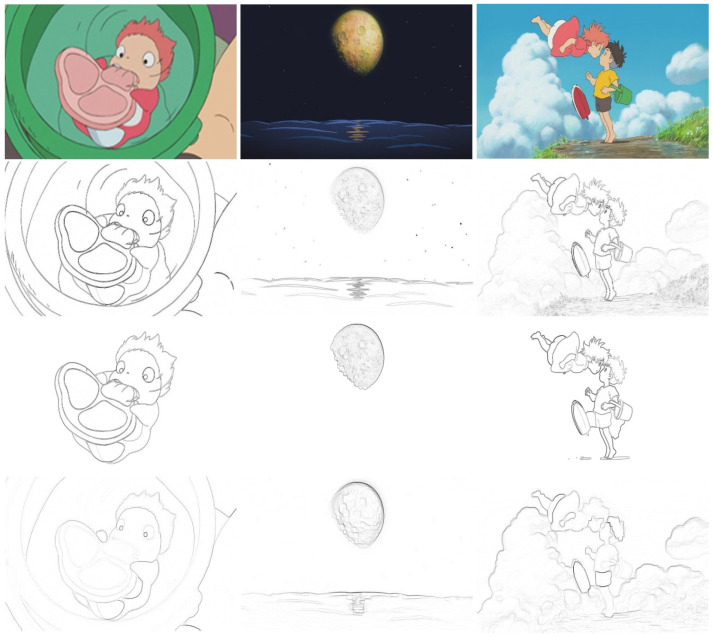
Edge detection results obtained using Sobel edge detection. Images shown on the top row are input content images, on the second row are the edge maps of the input content images, on the third row are the edge maps of the images with segmented main objects, and on the bottom row are the combined edge maps of the three smoothed images using the Sobel operator.

If the gradient magnitude at a pixel exceeds a user-defined threshold, this pixel is set as an edge point. Figure 4 shows the resulting images obtained using Sobel edge detection. Images shown on the top row are input content images and on the second row are the edge maps of the input content images, on the third row are the edge maps of the images with segmented main objects, and on the bottom row are the combined edge maps of the three smoothed images using the Sobel operator, respectively.

### 3.4. Sand Painting Generation

After completing the procedures above, we obtained five images, including the original content image, the main object image, and the three smoothed images with the corresponding edge maps. Subsequently, we performed neural style transfer to transfer these images and obtained five stylized sand painting images based on the edge maps and the input style image. Finally, we combined the five stylized images with different weights and obtained a smooth sand paint-stylized image that emphasized the main objects.

## 4. Results and Discussion

In this section, we describe several experiments conducted to evaluate the effectiveness of the proposed approach. Here, we describe the implementation details, content images, and style images of the experiment. In addition, we present the sand painting generation results yielded by the proposed approach and compare them with the results of a previous method [3]. Our approach was implemented on a PC with an Intel Core i7-8700 CPU 3.2 GHz and an NVIDIA GeForce GTX 1660 Ti. All experiments were performed using TensorFlow and CUDA 11.4. The L-BFGS algorithm [24] was used to train the neural network, and each stylized image was trained for 10 epochs. The content, style, and stylized image sizes were 925 × 500, 700 × 392, and 740 × 400 pixels, respectively. Moreover, it took about 1.5 min to generate a styled sand painting using the proposed approach. 

**Figure 5:**
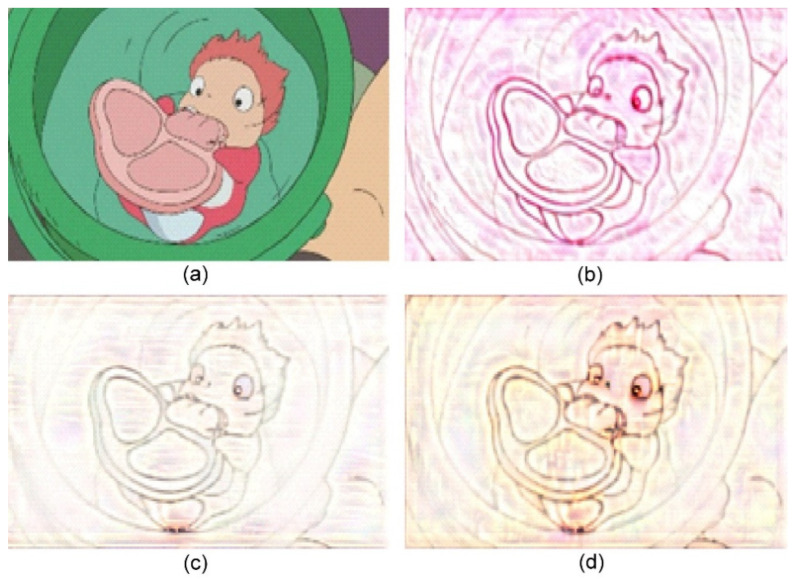
Sand painting generation using our approach. (**a**) Content image; (**b**) Stylized sand painting using style image from [26]; (**c**) Stylized sand painting using style image from [27]; and (**d**) Stylized sand painting using style image from [28].

**Figure 6:**
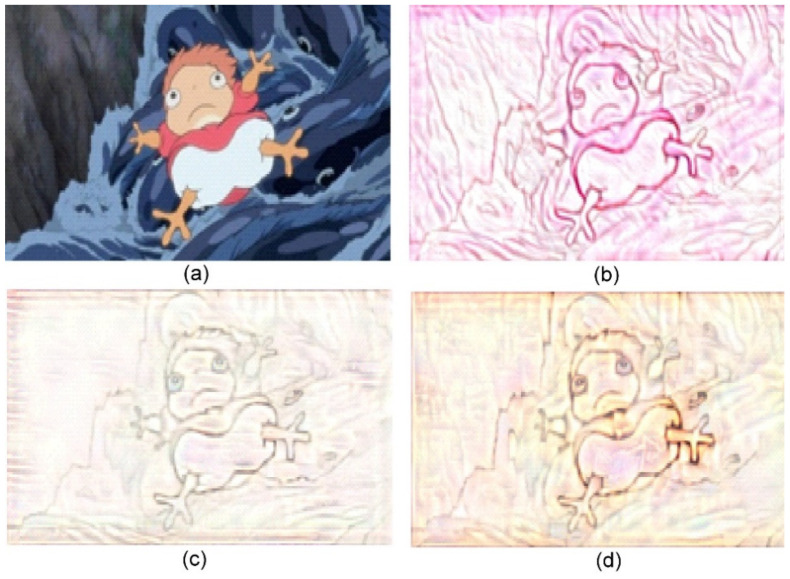
Sand painting generation using our approach. (**a**) Content image; (**b**) Stylized sand painting using style image from [26]; (**c**) Stylized sand painting using style image from [27]; and (**d**) Stylized sand painting using style image from [28].

**Figure 7:**
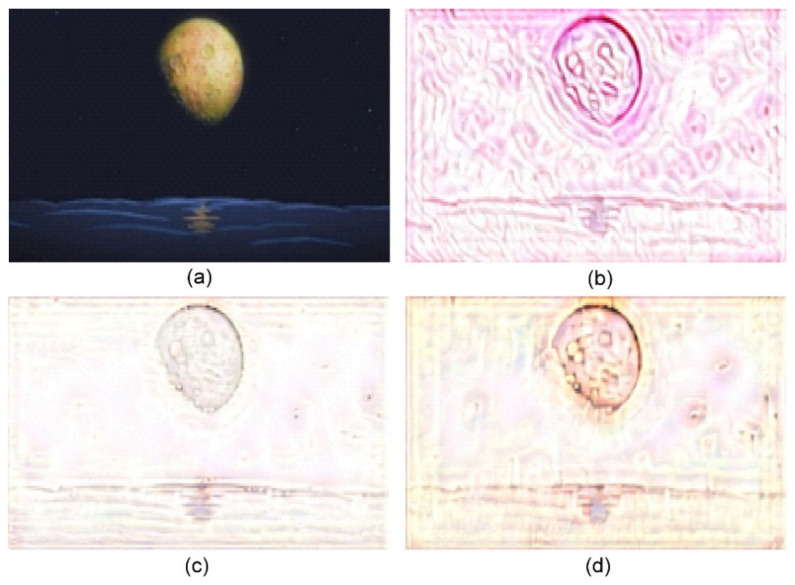
Sand painting generation using our approach. (**a**) Content image; (**b**) Stylized sand painting using style image from [26]; (**c**) Stylized sand painting using style image from [27]; and (**d**) Stylized sand painting using style image from [28].

**Figure 8:**
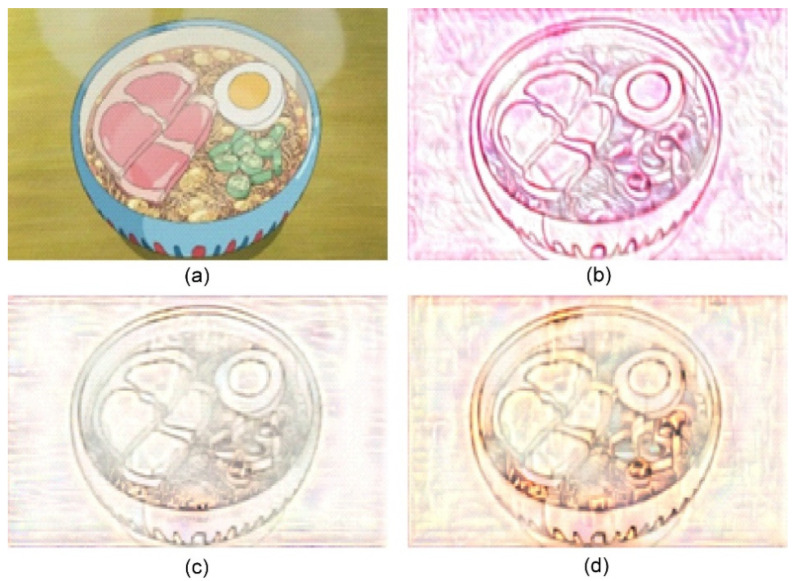
Sand painting generation using our approach. (**a**) Content image; (**b**) Stylized sand painting using style image from [26]; (**c**) Stylized sand painting using style image from [27]; and (**d**) Stylized sand painting using style image from [28].

**Figure 9:**
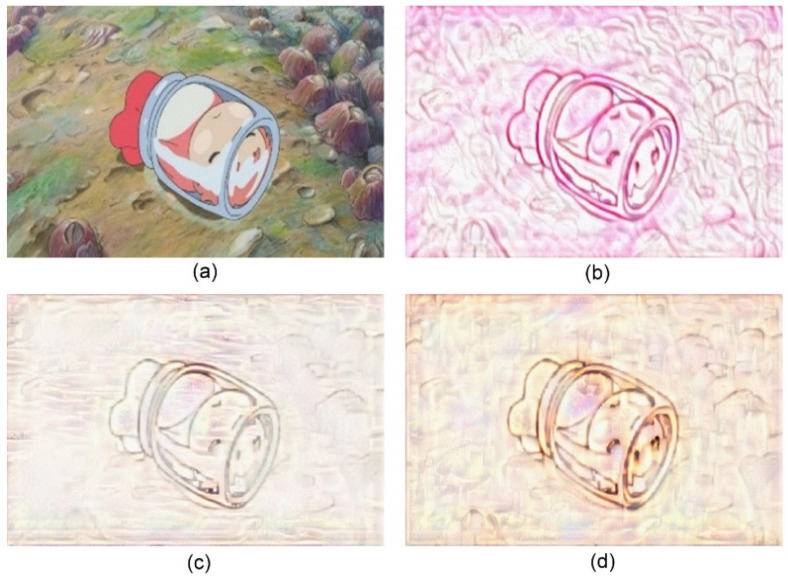
Sand painting generation using our approach. (**a**) Content image; (**b**) Stylized sand painting using style image from [26]; (**c**) Stylized sand painting using style image from [27]; and (**d**) Stylized sand painting using style image from [28].

**Figure 10:**
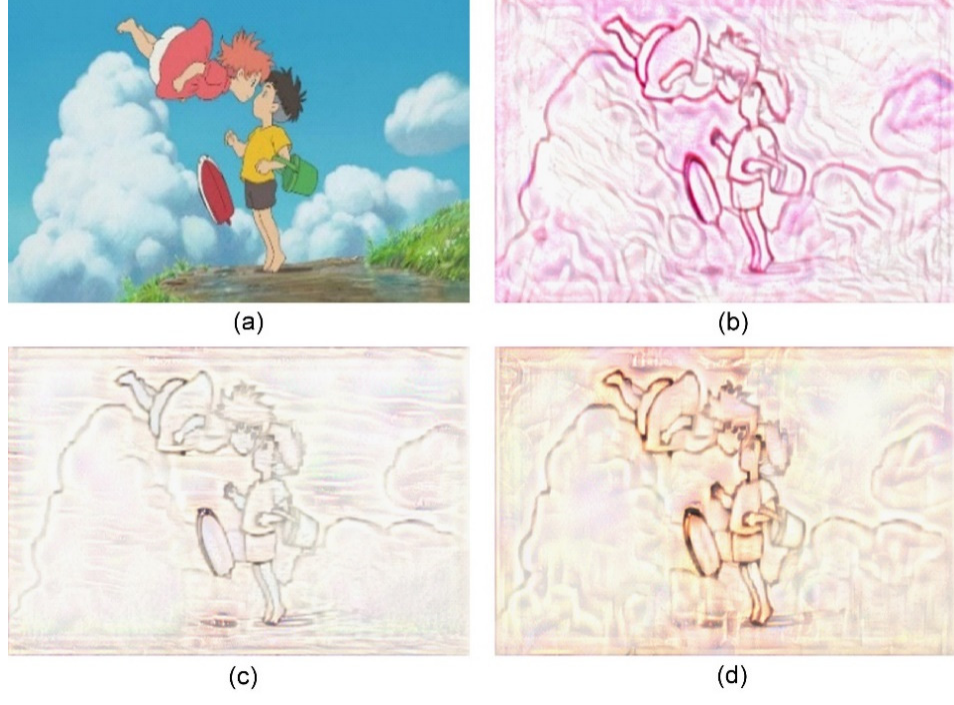
Sand painting generation using our approach. (**a**) Content image; (**b**) Stylized sand painting using style image from [26]; (**c**) Stylized sand painting using style image from [27]; and (**d**) Stylized sand painting using style image from [28].

The proposed approach can be applied to different types of content and style images. Without loss of generality, we selected one type of content images and three types of style images for the experiments. If other types of content images contained simple structures and clear edges, they are suitable for the transfer of the sand painting style using the proposed approach. We selected several images from the animated film “Ponyo” as the content images [25]. Additionally, we selected three images from the sand painting work “I Love My Home”, “Love”, and “Don’t be late” as the style images [26,27,28]. They contained distinct strokes and naturally scattered sandy backgrounds. These images were suitable as style images for sand painting style transfer. The sand paintings yielded in our approach using the three style images are presented in Figure 5, Figure 6, Figure 7, Figure 8, Figure 9 and Figure 10. The results show that our approach can preserve the details of the main objects, improve the stylized rendering of the sand paintings, and reduce the background noise. Hence, our approach can yield favorable visual results for sand paintings.

**Figure 11:**
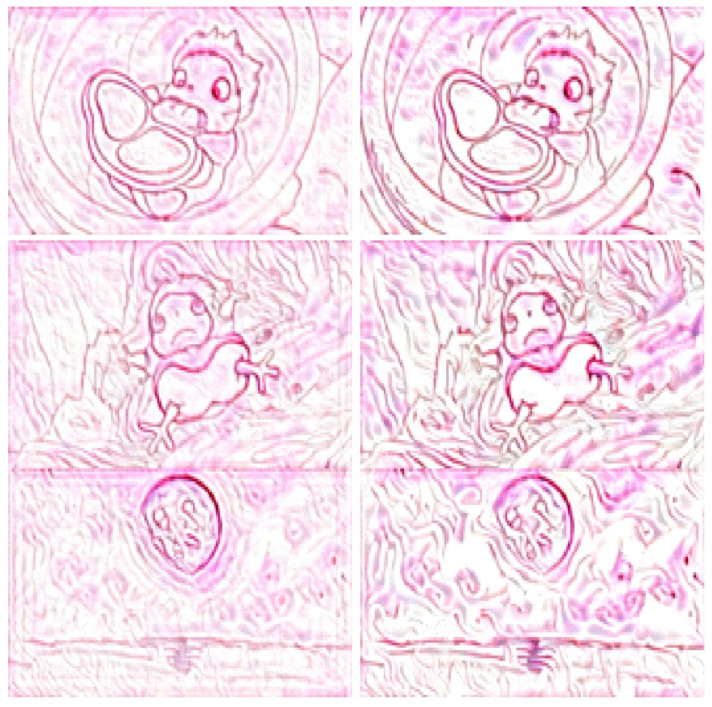
Comparison of sand painting generation. Images shown on left and right are stylized images generated using our approach and a previous method, respectively.

**Figure 12:**
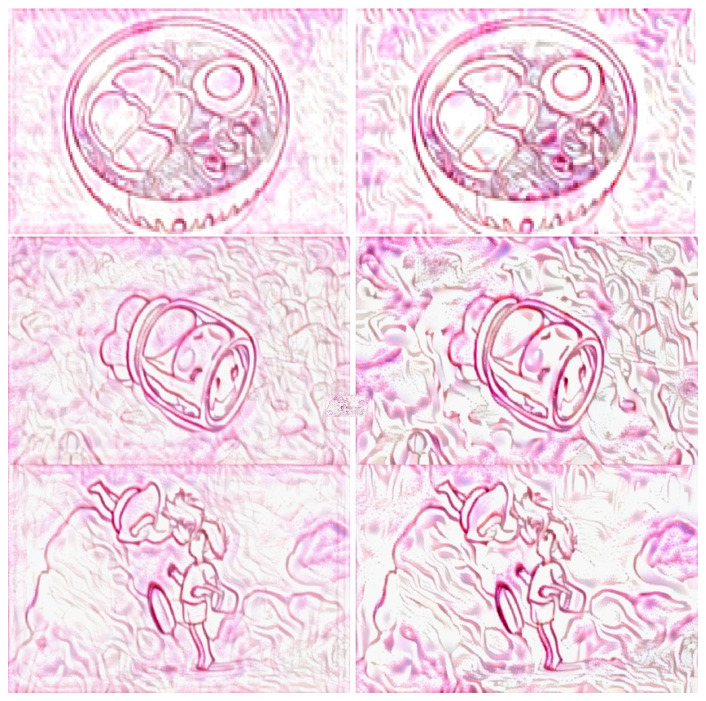
Comparison of sand painting generation. Images shown on left and right are stylized images generated using our approach and a previous method, respectively.

We compared the proposed approach with a previous method [3], and the results are shown in Figure 11 and Figure 12. In these figures, the images on the left and right show the stylized images generated using our approach and those generated using the previous method, respectively. Based on these images, the proposed approach retained the details of the main objects and improved the stylized rendering of the sand paintings. In addition, it reduced background noise caused by the neural style transfer. Hence, the proposed approach can effectively emphasize the main objects and optimize the presentation effect of sand painting generation using neural style transfer.

Finally, all styled sand painting images in Figure 11 and Figure 12 generated using our approach and the previous method [3] were evaluated qualitatively by 25 human evaluators. The evaluators subjectively assessed each styled sand painting image generated using our approach and the previous method [3] by subjectively assigning a score from 1 (poor) to 5 (good). The average scores of the styled sand painting images for the approaches are listed in Table 1. The average score for the proposed approach was 3.07, whereas that for the previous method was 2.36, which implies that the proposed approach performed better than the previous method.

The limitations of our approach are as follows:

- In the experiments, we selected one type of content image and three types of style image for the evaluation of our approach. This limits the scope of the experiments for evaluating the proposed approach.
- Our approach required approximately 1.5 min to generate a styled sand painting, which is considered a long duration, and thus is unsuitable for real-time interactive applications.

## 5. Conclusions

We proposed an automatic approach for sand painting generation using neural style transfer. The proposed approach retained the details of the main objects and improved the stylized rendering of sand paintings. Furthermore, it reduced the background noise caused by the neural style transfer. Compared with the previous method, which does not emphasize the main objects, our approach can obtain better visual effects of sand painting. Although the proposed method successfully obtained the desired effect and reduced noise, it could be unable to achieve a complete imitation of the sand painting effect. In future studies, we will evaluate whether the proposed approach can be applied to other art styles and whether it is suitable for other types of content images. Moreover, we will attempt to use different neural network training algorithms to assess whether they can achieve better transfer effects.

## Figures and Tables

**Table 1:** Qualitative evaluation.

	Average Score
Our approach	3.07
Previous method [3]	2.36

## Data Availability

The data presented in this study are openly available in [25].
